# Anterior Approach in a Huge Lipomatous Tumor of the Thigh

**DOI:** 10.1155/2014/839397

**Published:** 2014-10-01

**Authors:** Jordi Faig-Martí

**Affiliations:** Servei d'Ortopèdia, Hospital Sant Rafael, Passeig Vall d'Hebron 107-117, 08035 Barcelona, Spain

## Abstract

Confronted with a huge lipomatous anterior thigh tumor, the surgical approach had to be assessed. Those described in the literature did not seem appropriate for our case so some modifications were made. We present the case of a 77-year-old woman who presented with a huge anterior thigh compartment tumor with one-year evolution. Magnetic resonance imaging informed the presence of a lipomatous tumor with a possible vascular contact. Based on Thomson's anterior approach, but modifying the skin incision, the medial distal femur was reached until the neurovascular bundle and, proximally, the lesser trochanter. The tumor was totally resected due to a good visualization using this approach.

## 1. Introduction

Large lipomas and low-grade liposarcomas are occasionally met in our clinical practice and can raise some doubts in their treatment [1]. They are usually painless lesions of very slow growth with a typical appearance on magnetic resonance imaging (MRI) [2].

## 2. Case Report

**Figure 1:**
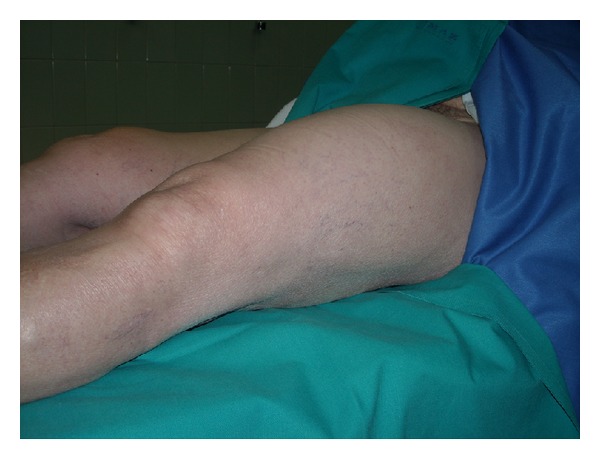
Enlargement of the left thigh.

**Figure 2:**
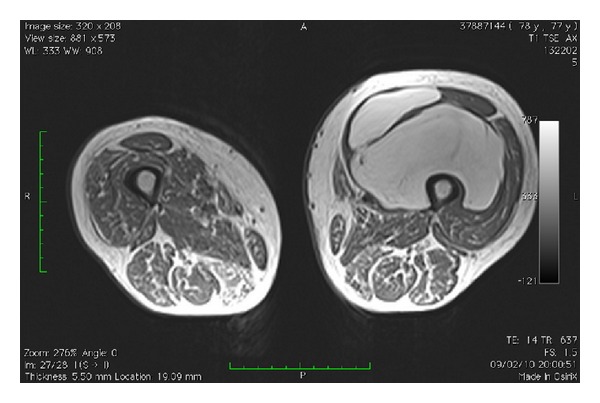
MRI of a lipomatous tumor in the anterior compartment of the thigh.

**Figure 3:**
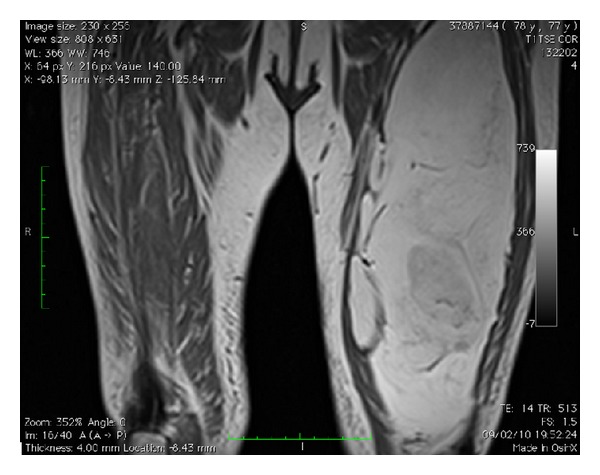
MRI of a lipomatous tumor in the anterior compartment of the thigh.

We report the case of a 77-year-old female who complained of a progressively enlarged left thigh in the last year (Figure 1). MRI showed the presence of a hyperintense mass on T1 and T2 with suppression in T2 fat suppression and STIR projections. The radiologist reported “thigh deep lipoma in contact with the femoral vessels in the medial thigh” (Figures 2 and 3).

**Figure 4:**
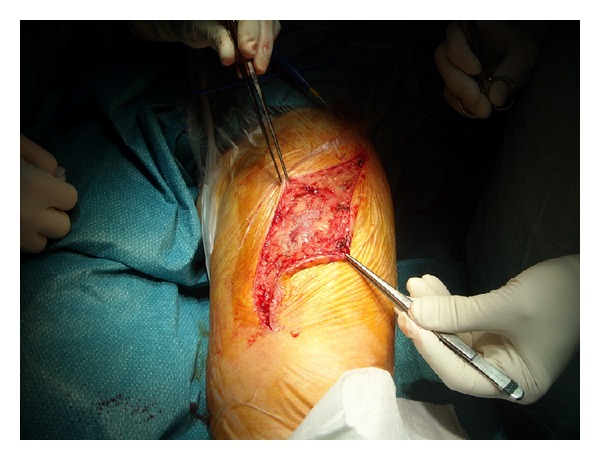
Anterior thigh S shaped incision.

**Figure 5:**
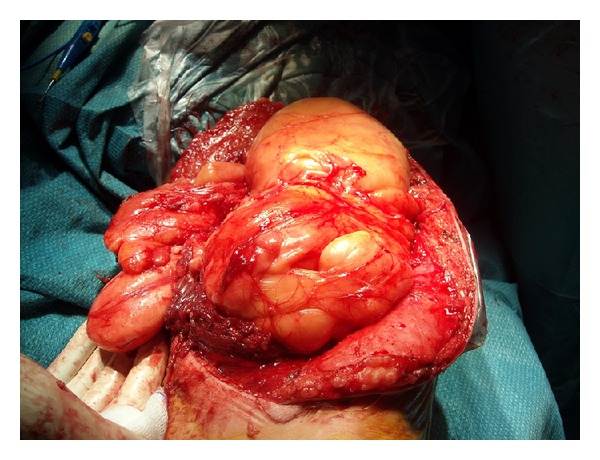
Tumor dissection between the rectus anterior and vastus lateralis.

**Figure 6:**
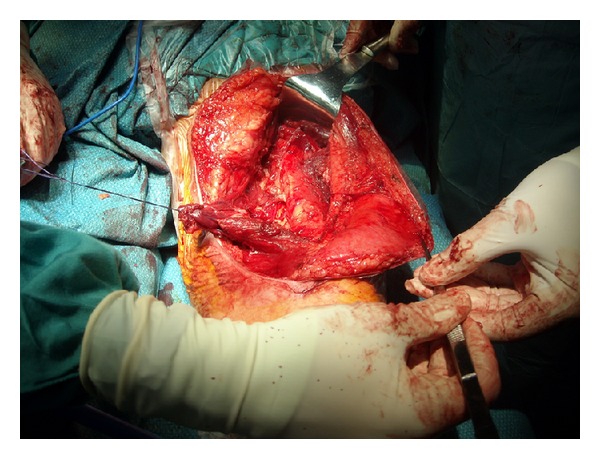
Flap of the distal insertion of the vastus medialis and medial aspect of the intermuscular septum after resection of the tumor.

**Figure 7:**
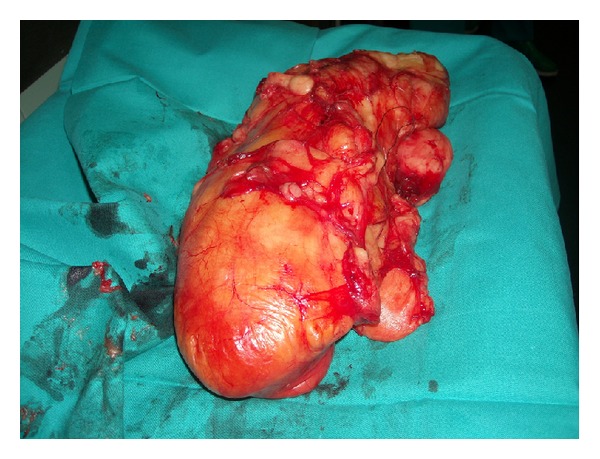
Macroscopic appearance of the resected tumor.

To achieve a good access both medially and externally on the thigh and avoid traction on the ends of the incision, an S shaped skin incision was performed on the anterior thigh approximately along the direction of the sartorius muscle (Figure 4). Following the outer edge of the rectus anterior, the tumor was located below this muscle and both muscle and tumor were separated by blunt dissection with the finger until the distal insertion of the vastus lateralis (Figure 5). At this level, the dissection was continued along the medial side of the rectus, sectioning the vastus medialis insertion but leaving enough muscle to allow for reinsertion [3]. This medial approach allowed a good access to the medial intermuscular septum, where possible surgical vascular injury may have required a vascular suture (Figure 6). At this level we find the adductor magnus and below it the tibial nerve and popliteal artery and vein. Dissection between the tumor and the medial intermuscular septum caused no vascular injury and a lipomatous tumor weighing 2500 g formed by two masses measuring 16 × 9 cm and 26 × 13 cm could be completely removed (Figure 7). Histopathological examination reported that it was a low-grade or well-differentiated liposarcoma.

Postoperatively, the patient had difficulties in flexion and extension of the hip and knee, due to muscular atrophy she presented preoperatively. Therefore, a physical therapy program aimed at increasing the range of motion and muscle power of the hip and knee was initiated. Both parameters were normalized at the control visit at two months. At 12 months the patient was asymptomatic, with no signs or symptoms of recurrence, as well as at four-year followup.

## 3. Discussion

Lipomas and liposarcomas are tumors of mesenchymal origin with adipose cells. Liposarcomas include 10–16% of all soft tissue sarcomas [4] and include five subtypes with different morphological, cytogenetic, and clinical features. One of them is atypical lipomatous tumor or well-differentiated liposarcoma. It is a locally aggressive lipomatous tumor without metastatic potential, particularly common in the thigh, although at this location degeneration risk is low and stands at 2% [5]. In some liposarcomas, radiotherapy may be indicated pre- or postoperatively. However, some authors consider that it is not necessary in cases of complete resection and histological diagnosis of well-differentiated liposarcoma as was our case [6, 7].

In the treatment of these tumors, the surgeon should be aware of the possibility of degeneration, even to a low-grade malignancy, but requiring complete excision to prevent recurrence. MRI may help in differential diagnosis [8]. Due to the large size of the tumor and its proximity to the medial thigh vessels in our case, the question of the best approach for a safe and complete tumor resection arose. Some orthopedic surgeons are more comfortable following external approaches to the thigh that can reach the entire femur. But this type of approach has the disadvantage of leaving the medial vascular structures out of sight that may be injured while trying to dissect them if the tumor is very close. Therefore an anterior thigh approach was performed in the present case.

McRae [9] described (following Henry and Thomson) the anterolateral approach to the femoral shaft using a straight skin incision line and the interval between the outer edge of the rectus muscle and the medial border of the vastus lateralis, after sectioning the vastus intermedius parallel to its fibers to access the femur. The main drawback reported in this approach is postoperative quadriceps adhesions that make it rarely be indicated.

The main dangers of the approach used include injury to the superior medial geniculate artery which must be ligated or coagulated. The popliteal vessels are protected by the adductor magnus unless local dissemination of the process reaches them. In the proximal incision, if we get too close to the hip, the nerve of vastus lateralis and the lateral femoral circumflex artery can be injured. This artery emerges from the deep femoral artery and runs along the outer edge of the rectus muscle proximally. If necessary it can also be ligated.

The approach can be extended proximally along the interval between the sartorius and tensor fasciae latae in what would be an anterior approach to the hip joint. Distally, the approach can continue with a medial parapatellar approach to the knee.

## 4. Conclusion

The surgical approach used in the case presented allowed a good visualization of the entire tumor bed which resulted in a complete removal without any local or systemic complications. The subsequent rehabilitation has overcome muscle atrophy and muscle adhesions reaching symmetrical limb functionality. The aesthetic appearance of a long S curved incision compared to a straight incision of the same length can be a determining factor to be considered in younger patients who may be concerned about the scar appearance.
